# Prospective assessment of early developmental markers and their association with neuropsychological impairment

**DOI:** 10.1007/s00431-023-05182-y

**Published:** 2023-09-14

**Authors:** Elisa Cainelli, Luca Vedovelli, Daniele Trevisanuto, Agnese Suppiej, Patrizia Bisiacchi

**Affiliations:** 1 Department of General Psychology, University of Padova, Via Venezia, 8 – 35133 Padova, Italy; 2 Unit of Biostatistics, Epidemiology, and Public Health, Department of Cardiac, Thoracic, Vascular Sciences, and Public Health, University of Padova, Padova, Italy; 3 Department of Woman and Child Health, University Hospital of Padova, Padova, Italy; 4 Department of Medical Sciences, Pediatric Section, University of Ferrara, Ferrara, Italy; 5 Padova Neuroscience Centre, PNC, Padova, Italy

**Keywords:** Neonatal, Psychomotor development, Griffiths Mental Developmental Scale, Perinatal asphyxia, Hypoxic ischemic encephalopathy, Preterm

## Abstract

Children who experience adversities in the pre-perinatal period are at increased risk of developing impairment later in life, despite the absence of overt brain and neurological abnormalities. However, many of these children exhibit sequelae several years after a period of normal appearance. As a result, the need for reliable developmental assessments for the early detection of infants at high risk of adverse neurodevelopmental outcomes has emerged. The Griffiths Mental Developmental Scales have a promising but poorly explored prognostic ability. This longitudinal study evaluated the predictive power of the Griffiths Mental Developmental Scales at 12 and 24 months on the cognitive and neuropsychological profile at 6 years of age in a sample of 70 children with a history of prematurity or perinatal asphyxia but without brain and neurological abnormalities. We found that the Griffiths Mental Developmental Scales at 24 months had good predictive ability on the intelligence quotient at 6 years and the capacity to predict some neuropsychological performances. On the other hand, the Griffiths Mental Developmental Scale at 12 months was not associated with the performance at 6 years or 24 months.

*Conclusion*: Data on brain development converge to indicate that the first two years of age represent a critical stage of development, particularly for children experiencing mild pre-perinatal adversities who are thought to exhibit white matter dysmaturity. For this reason, this age is crucial for identifying which children are at major risk, leaving enough time to intervene before overt deficits become apparent. Brain development in the first 2 years could explain the limited reliability of early neurodevelopmental testing.

**Table:** 

**What is Known:** *• Pre-perinatal adversities increase the risk of developing neurodevelopmental disorders.* *• The predictive ability of the Griffith scale is poorly explored in low-grade conditions.*	
**What is New:** *• The predictive ability of the Griffith scale has been investigated in low-risk children.* *• A complete neuropsychological profile could offer a more accurate prediction than the intellectual quotient.*

**Supplementary Information:**

The online version contains Supplementary material available at 10.1007/s00431-023-05182-y.

## Introduction

Clinicians are faced with the challenge of identifying early indicators of risk during the developmental period, especially in the presence of certain pathological conditions that may have a delayed impact. The development of cognition, personality, and behavior is a slow process, and outcomes may take years to emerge. Brain plasticity is also at its maximum during early development, and its ability to reorganize after adversity is unpredictable. These challenges are particularly relevant when considering children who have experienced pre-perinatal adversities, which occur during a critical phase of brain maturation [1, 2]. Brain distress in infants can lead to early structural and neuromotor dysfunctions, but subclinical cerebral reorganization is often undetectable with standard instruments. Infants with normal magnetic resonance imaging, mildly abnormal electroencephalogram, and no pathological clinical signs are typically considered low-risk and may not receive long-term follow-up. However, studies have shown that impairments can develop over time and with varying severity [3, 4]. Research on neonatal risk indicators provides promising insights, but evidence remains limited. Although experimental diagnostic techniques, such as quantitative neurophysiology and biochemistry, are promising, they are still far from being included in clinical routine [4–6].

The need for accurate developmental assessments for early detection of infants at high-risk for adverse neurodevelopmental outcomes has emerged. The Griffiths Mental Developmental Scales (GMDS) [7] is a well-recognized instrument, but its prognostic role is not well-established. A tool with early predictive value is crucial due to the time needed for some neuropsychological impairments to emerge. Prematurity and hypoxic ischemic encephalopathy have a relevant epidemiological incidence, and the predictive value of early assessments has been evaluated for these conditions [8–14]. Furthermore, a few studies have been done on healthy children [15, 16].

Our study aimed to assess the ability of the GMDS at 12 and 24 months to predict the cognitive and neuropsychological profile of children at high risk of developing neurodevelopmental impairments, specifically premature birth and hypoxic ischemic encephalopathy, but who were considered "apparently healthy" without any signs of magnetic resonance imaging or electroencephalographic abnormalities or neurological issues.

## Methods

### Participants

We utilized data from two ongoing projects that followed newborns admitted to the neonatal intensive care unit due to preterm birth or neonatal hypoxic ischemic encephalopathy. The study was conducted in accordance with the Code of Ethics of the World Medical Association (Declaration of Helsinki) for experiments involving human subjects. It was approved by the hospital's ethics committee. Parents provided written informed consent for their children to participate. The children were recruited from January 2010 to June 2015, and the neonatal inclusion and exclusion criteria are reported in the Supplementary material.

Neonatal clinical characteristics of preterm and hypoxic ischemic encephalopathy children are reported in Supplementary material, Tables S1 and S2.

Children in the study received a standardized follow-up program that included a psychomotor developmental assessment using the GMDS at 12 and 24 months, as well as a cognitive and neuropsychological evaluation at 6 years. Age was adjusted for gestational age at birth for preterm children up to 24 months, and all children underwent neurological examinations at the same time.

Excluded from this substudy were children with neurological and brain abnormalities shown by magnetic resonance imaging or ultrasonography, neurosensorial impairments, congenital malformations, inborn errors of metabolism, genetic syndromes, other medical comorbidities, traumatic events or documented parental neglect, and invalidating parental pathologies or behaviors. The final sample consisted of 70 children (40 with a history of hypoxic ischemic encephalopathy and 30 with prematurity), 29 females and 41 males. Age at first GMDS evaluation was 12 ± 1 month, at the second 24 ± 1.5 month, at the neuropsychological assessment 6.3 ± 0.8 years.

### GMDS evaluation

We used GMDS 0–2, 1996 revision [7], to obtain standardized developmental assessments. The scales were scientifically constructed based on item analysis and developmental theory; these scales were chosen for their clinical utility, psychometric properties, and validity. The GMDS provided a simple ratio transformation, dividing the mental age by chronological age, yielding means and standard deviations for the global quotient and the five subscale scores: Locomotor, Personal-social, Language, Eye-hand Coordination, Performance tests [16].

### Neuropsychological assessment

The same examiner also measured general intelligence using the Wechsler Preschool and Primary Scale of Intelligence III (WPPSI-III) [17] test or the Wechsler Intelligence Scale for Children IV (WISC-IV) [18], standardized for the Italian population.

The neuropsychological profile was measured using the following: the Naming test for language [19]; for attention, the Visual and Auditory Attention tests of the NEPSY-II [20]; for memory, the Digit Span and the Corsi tests, which evaluate the short-term verbal and visual-spatial memory, and the Word's List and Word’s Recall tests, which evaluate the learning of verbal material and long-term retrieving abilities [19]; for executive function, the Coding test of the WISC-IV or WPPSI-III [17, 18] and the semantic verbal Fluency test, which evaluates the ability to access the lexicon through a categorical cue [19]; and for social skills, the Affect Recognition test of the NEPSY-II [20].

### Risk factors

A structured interview and questionnaire (the Parent Stress Index-Short Form) [21] were performed to establish the presence of environmental risk factors that could significantly influence neurodevelopment and consequently represent a confounding factor [22]. A risk factor index was calculated (see Supplementary materials for the procedure).

### Statistical analysis

Data are expressed as mean ± standard deviation or percentage, according to the variable type. Statistical analyses were carried out with R statistics, version 4.2.2 (R Core Team, 2021. R: A language and environment for statistical computing. R Foundation for Statistical Computing, Vienna, Austria. https://www.R-project.org/).

#### Missing data

We visually inspected missing data patterns and then applied multiple imputations using multivariate imputation by the chained equations approach and the {mice} R package [23]. We imputed 13 datasets (according to the mean percentage of missingness) with 10 iterations and a random forest-based imputation method. All of the subsequent analyses were conducted with the pooled imputed dataset.

#### Neuropsychology at 6 years

To investigate the relationship between the neuropsychological variables measured at 6 years and the GMDS global quotients scores obtained at 12 and 24 months, we employed a linear model that controlled for the presence of risk factors and the disease group. This allowed us to examine how the GMDS scores predicted the profile at 6 years. Additionally, we used the same model structure to assess the association between the neuropsychology variables at 6 years and the GMDS subscales measured at 24 months. To account for the ordinal nature of the auditory attention variable, we fitted an ordinal regression model instead of the linear model.

#### Concordance between 12 and 24 months

We assessed the concordance between GMDS scores at 12 and 24 months using the global intraclass correlation coefficient with a two-way random effects model and a single rater/measurement consistency definition. We also used a multilevel linear model to analyze the longitudinal changes in GMDS scores over time and across subtests. The model included the GMDS score as the dependent variable and time and subtest as predictors. The interaction term between time and subtest allowed for different changes across time and subtests. A random intercept for each participant accounted for individual variability in GMDS scores.

#### GMDS at 12 and 24 months concordance

A qualitative method was employed to assess the trend of the GMDS scores. Concordance between two global quotients (at 12 and 24 months, at 12 months and 6 years, and at 24 months and 6 years) was evaluated considering 1 standard deviation (i.e., 15 points variation). The performance was considered improved or worsened if the difference between two global quotients exceeded or lacked 15 scores. Conversely, if the difference was less than 15 points, the global quotient was considered stable over time.

## Results

Forty infants with hypoxic-ischemic encephalopathy (23 males, 17 females) and 30 with prematurity (18 males, 12 females) were included (Supplementary Tables 1 and 2). No loss to follow-up occurred.

The scores obtained at the 12 and 24 months and 6 years evaluations are reported in Tables 1 and 2, both for the whole group and the two subgroups (preterm and hypoxic ischemic encephalopathy). For global quotient at 24 months, we found a significant association with intelligence quotient (p < 0.001), semantic fluency (p < 0.001), and Word’s List (p = 0.044). Conversely, we did not find evidence for an association between intelligence quotient and neuropsychological scores (language, attention, memory, executive functions and social skills) measured at 6 years and GMDS global quotient measured at 12 months, disease group (premature or hypoxic ischemic encephalopathy), and risk factors index. The concordance assessment showed that each GMDS subscale was significantly different from 12 to 24 months (intraclass correlation coefficient = 0.30; Table S3).

**Table 1:** GMDS percentage of impaired scores (< 1.5 SD) obtained at 12 and 24 months

	**Group, N = 70**	**Preterm, N = 30**	**HIE, N = 40**
**GMDS 12 months**			
**Global Quotient**	1 (1%)	0 (0%)	1 (2%)
**Locomotor A**	6 (8%)	1 (3%)	5 (12%)
**Personal-social B**	3 (4%)	2 (6%)	1 (2%)
**Language C**	1 (1%)	0 (0%)	1 (2%)
**Eye-hand coordination D**	4 (6%)	2 (6%)	2 (5%)
**Performance E**	0 (0%)	0 (0%)	0 (0%)
**GMDS 24 months**			
**Global Quotient**	10 (14%)	4 (12%)	6 (15%)
**Locomotor A**	7 (10%)	2 (6%)	5 (12%)
**Personal-social B**	3 (4%)	1 (3%)	2 (5%)
**Language C**	11 (16%)	5 (16%)	6 (15%)
**Eye-hand coordination D**	9 (13%)	3 (10%)	6 (15%)
**Performance E**	23 (33%)	5 (16%)	18 (45%)

*HIE* hypoxic ischemic encephalopathy

**Table 2:** Cognitive and neuropsychological percentage of impaired scores (< 1.5 SD) obtained at the 6-year follow-up

**Test**	**Group, N = 70**	**Preterm, N = 30**	**HIE, N = 40**
Intelligence Quotient	7 (10%)	2 (6%)	5 (12%)
Coding	9 (13%)	4 (13%)	5 (12%)
Semantic Fluency	14 (20%)	6 (20%)	8 (20%)
Naming	5 (7%)	1 (3%)	4 (10%)
Words list	3 (4%)	1 (3%)	2 (5%)
Recall list	6 (8%)	2 (6%)	4 (10%)
Corsi	1 (1%)	0 (0%)	1 (2%)
Digit Span	3 (4%)	1 (3%)	2 (5%)
Visual attention	2 (3%)	0 (0%)	2 (5%)
Auditory Attention (< 10th percentile)	23 (33%)	11 (36%)	11 (36%)
Affect recognition	12 (17%)	5 (16%)	7 (17%)

*HIE* hypoxic ischemic encephalopathy

**Fig. 1:**
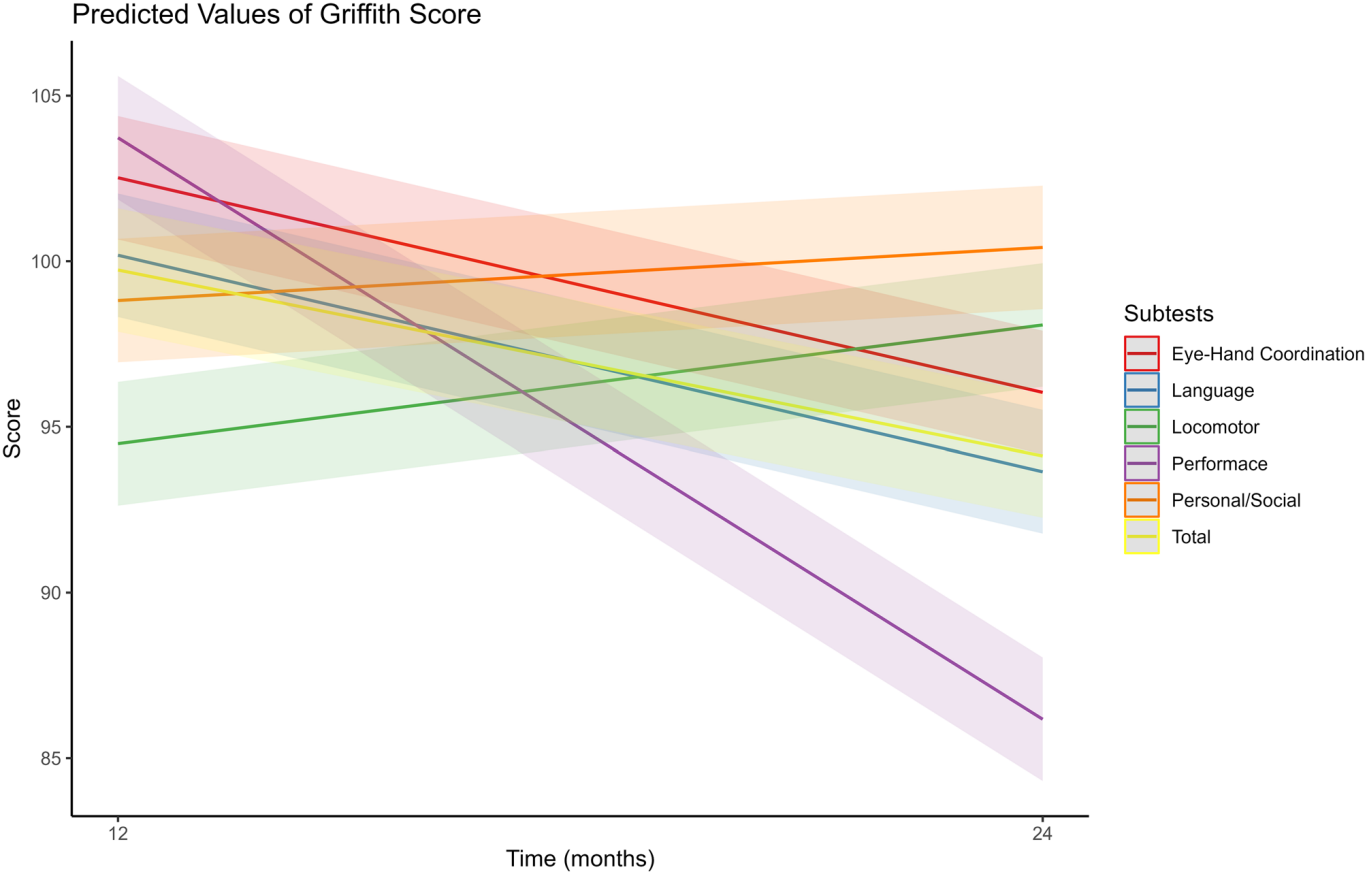
Estimated GMDS scores at 12 and 24 months for each subtest, showing overall change over time and consistent patterns of change between the two timepoints. We represented the change of predicted score (estimated using the model presented in Table S3) for each subtest (from **A** to **E**). Concordance between the two timepoint would have been represented by near-horizontal lines

Figure 1 displays predicted values of GMDS scores for each subtest at 12 and 24 months, generated using a multilevel linear model accounting for individual differences and time*subtest interaction. Scores increased for all subtests between 12 and 24 months, with Language and Performance showing the largest increase.

At 24 months, we examined the association of cognitive and neuropsychological scores with GMDS subscales and found the strongest association with intelligence quotient. Beta coefficients and additional results are reported in Table 3.

**Table 3:** At-glance associations between GMDS subtests at 24 months and neuropsychology at 6 years

**Association**	**Beta**	**95% CI**	**p-value**
**QI vs. 24 months subscale A**	0.37	0.05, 0.69	0.014
**QI vs. 24 months subscale B**	0.42	0.09, 0.77	0.014
**QI vs. 24 months subscale D**	0.58	0.20, 0.97	0.004
**QI vs. 24 months subscale E**	0.54	0.20, 0.89	0.003
**Semantic fluency vs. 24 months subscale B**	0.03	0.01, 0.05	0.003
**Semantic fluency vs. 24 months subscale C**	0.02	0.01, 0.04	0.019
**Semantic fluency vs. 24 months subscale D**	0.04	0.02, 0.06	< 0.001
**Semantic fluency vs. 24 months subscale E**	0.04	0.02, 0.06	< 0.001
**Word’s list vs. 24 months subscale D**	0.03	0.01, 0.06	0.002
**Word’s Recall vs. 24 months subscale D**	0.03	0.01, 0.06	0.005
**Visual attention vs. 24 months subscale D**	0.05	0.00, 0.11	0.032
**Auditory attention vs. 24 months subscale A**	0.10	0.02, 0.19	0.020
**Auditory attention vs. 24 months subscale B**	0.14	0.05, 0.23	0.002
**Auditory attention vs. 24 months subscale D**	0.10	0.00, 0.20	0.042

### GMDS at 12 and 24 months concordance

**Fig. 2:**
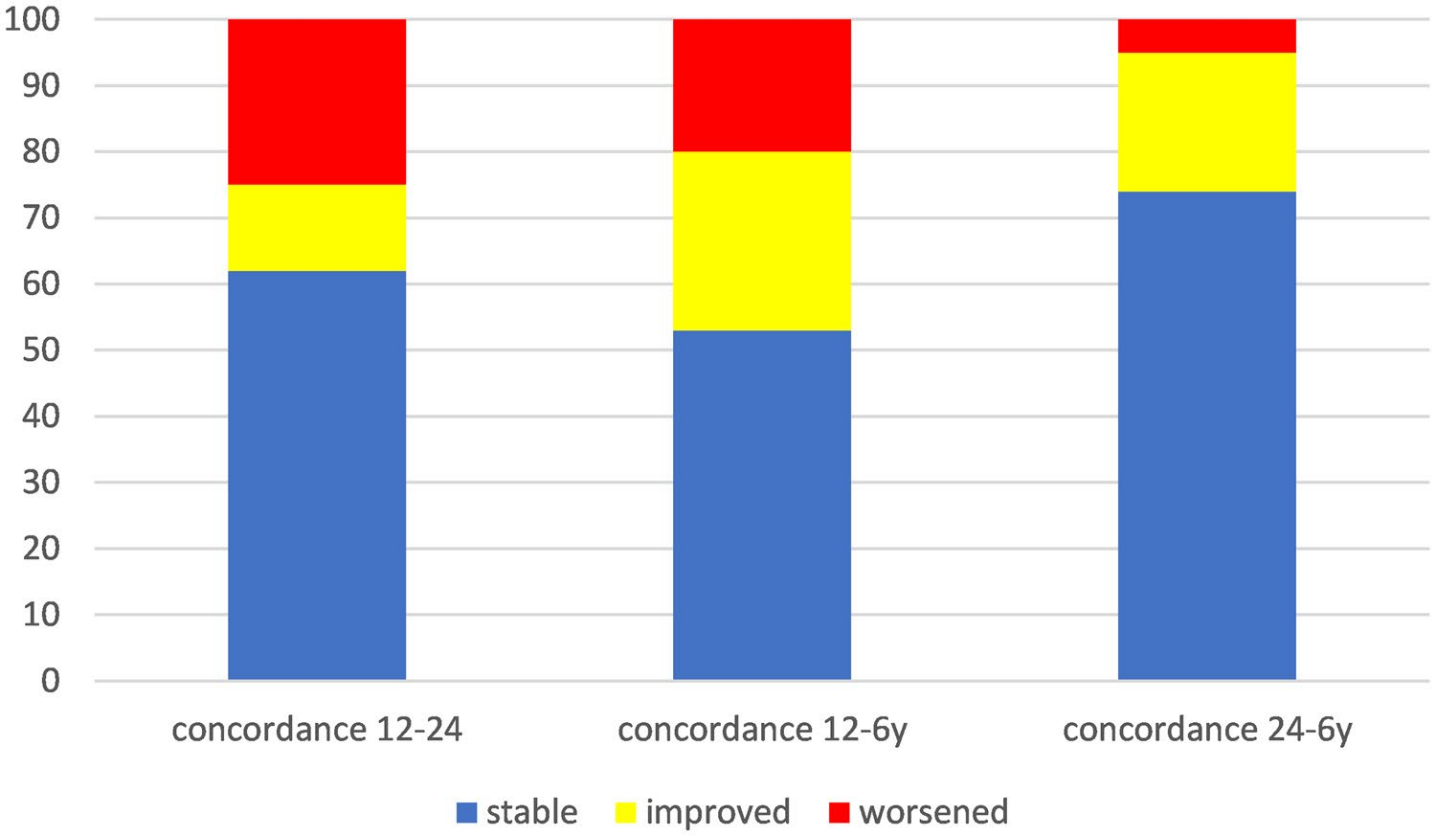
Percentage of children showing stable, improved, and worse performance over time in relation to the two assessments (12 and 24 months, 12 months and 6 years, 24 months and 6 years)

**Fig. 3:**
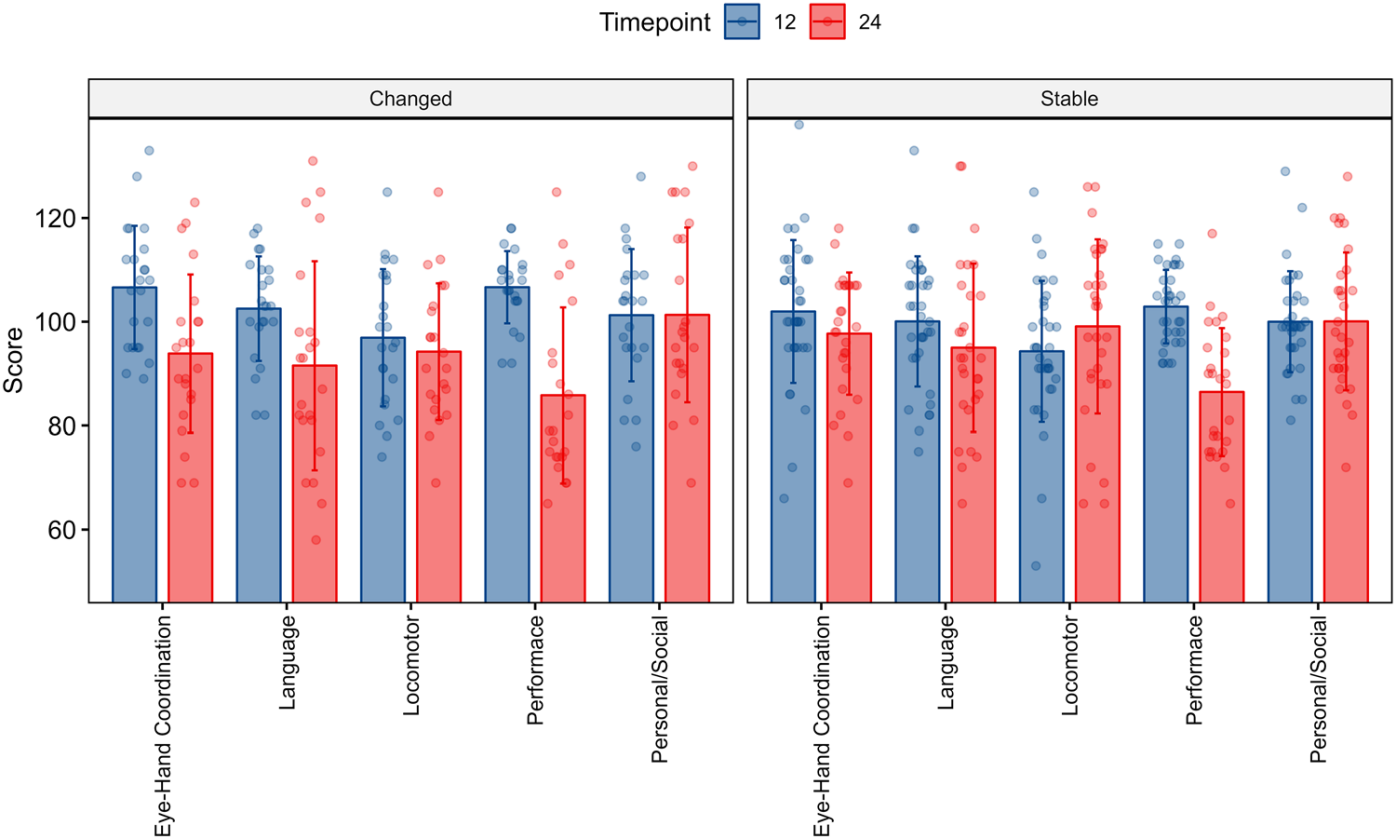
Comparison of GMDS subscales at 12 and 24 months showing stable performance and performance that changes over time. Mean values and standard deviations are displayed for each subscale, with blue representing the 24 months group and red representing the 12 months group. Language, Eye-hand coordination, and Performance appear to be the most variable subscales between the two time points

We assessed the concordance of the global quotient between two evaluations at different time intervals using the discrepancy of 1 standard deviation as the criterion (Fig. 2). The first evaluation was performed between 12 and 24 months, the second between 12 months and 6 years, and the third between 24 months and 6 years. The results showed that between 12 and 24 months, 62% of the children remained stable, 13% improved, and 25% worsened; between 12 months and 6 years, 53% remained stable, 27% improved, and 20% worsened. Between 24 months and 6 years, 74% of children remained stable, 21% improved, and 5% worsened. Figure 3 displays the specific trend of GMDS subscales at 12 and 24 months, stratified by children with globally stable performance and those who changed over time. The subscales with the most variable pattern over time were Language, Eye-hand Coordination, and Performance.

## Discussion

The present study aimed to investigate the predictive power of GMDS performed at 12 and 24 months on the cognitive and neuropsychological profile at 6 years of age in a sample of children with prematurity or hypoxic ischemic encephalopathy but without clinical or neurological dysfunction. The results suggest that GMDS performed at 24 months has a good predictive ability for the total intelligence quotient and some neuropsychological tasks (e.g., attention, language, and memory tasks) at 6 years of age. No significant association was found between the GMDS performed at 12 months and either the total intelligence quotient at 6 years or the global quotient at 24 months. These findings suggest that the GMDS performed at 24 months could be a useful tool for the early identification of developmental risks and early intervention in children with prematurity or hypoxic ischemic encephalopathy.

Some studies investigated the prognostic role of GMDS on the later cognitive outcome in healthy children [16] and children born with low weight [10, 14], premature [11, 13], or perinatal asphyxia [9, 12]. Most studies found a predictive ability of the GMDS before 24 months, but increasing at older ages.

Our study did not find any association between the GMDS score at 12 months and the cognitive and neuropsychological profile at 6 years of age. Furthermore, we did not observe any concordance between the scores obtained at 12 and 24 months. One possible explanation for this lack of concordance could be that we excluded children with neurological or clinical signs of dysfunctions, who often exhibit impairments early on. Including such children in future studies may enhance the predictive ability of the GMDS. However, our results raise doubts about its ability to detect impairments in this challenging group of asymptomatic children at risk of developing cognitive impairments very late. In a study with a similar focus, Romeo et al. [13] investigated the association between GMDS scores and later intelligence quotient in premature infants at low risk, albeit with less strict selection criteria than in our study. They found a correlation at 12 months and higher sensitivity and specificity at 24 months.

Our results demonstrate a scarce utility of performing GMDS at 12 months and raise whether this testing is justified and should still be recommended. However, such a strong decision should rely on at least two studies performed on independent populations. Indeed, other studies (performed in slightly different populations) found some utility already at 12 months [for example, 13], raising the question of whether the results we obtained may not be specific to the population (medical characteristics and regional specificities) included in our study so that such a strong conclusion cannot be drawn. Nevertheless, given these results, a need has emerged for further studies to elucidate whether GMDS testing at 12 months is still to be recommended in asymptomatic infants at risk of long-term developmental impairment.

When considering the whole continuum from pathological conditions to healthy children, an age higher than 2 years emerges as the best choice for identifying early reliable indicators of later risk. The first two years of life reflect a crucial phase in the brain's developmental process, where the fundamental structural and functional architecture is established. Subsequent maturation involves slower processes, such as reorganization, fine-tuning, plasticity, and remodeling of already established major circuits and networks. The first year of life sees a substantial grey matter volume increase, while white matter volume growth is slower and more prolonged [24]. However, most of the “wiring” of the brain is still present at birth, and the general pattern of adult myelination is present by the end of the second year [25]. After the second year, myelination does continue at a slower rate. Furthermore, after two years, the child becomes sufficiently interactive and able to express several ways of communication/interactions so that testing becomes more feasible, reliable, and reproducible.

In children with prematurity and hypoxic ischemic encephalopathy, despite heterogeneous manifestation, in the absence of overt brain damage, a process of white matter dysmaturation has been suggested [4]. This process is kindled by the surge of brain reactive oxygen species that impairs the precursors of myelin-forming cells during a precise susceptibility window [4]. The slower maturation rate of the myelination process may be a key factor in understanding our results. If the abnormalities in low-risk perinatal asphyxia and premature infants mainly affect the white matter, it would make sense that we would need to wait until 2 years to have reliable data. As we explained earlier, the myelination process progresses at a slower rate, and there is crucial advancement in the second year, unlike other brain processes that occur mainly in the first year. Therefore, the results of our study suggest that the GMDS at 24 months may be a useful tool for predicting cognitive and neuropsychological outcomes at 6 years of age in asymptomatic children at risk.

The present study provides novel insights into the predictive power of GMDS on the neuropsychological profile. Neuropsychological measures made it possible to detect more specific or subtle delays or deficits in development than global measures of cognitive status. The results suggest that attention and semantic fluency are associated with developmental scores at 24 months. These findings suggest that the GMDS scores may reflect cognitive milestones of later high-processing cognitive skills that request distributed neural networks such as attention.

One limitation of the present study is the heterogeneity of the sample in terms of medical history. However, previous studies of our group did not find specific developmental trajectories or neuropsychological phenotypes associated with a particular medical history in children with similar clinical characteristics [26, 27]. It was found that there was not enough evidence to support a difference between the two groups (premature and hypoxic ischemic encephalopathy). This suggests that generalizability to other, similar groups may be possible.

In conclusion, data on cortical or subcortical gray and white matter converge, indicating that 2 years is an appropriate time point for testing. For this reason, it takes about two years to become possible to identify whose children are at major risk while preserving enough time to intervene before the overt deficits become apparent. By contrast, most processes are already incomplete before 2 years, making earlier assessments much more variable and less sensitive. This appears particularly important for the challenging group of children with a history of pre-perinatal adversities but without structural or neurological impairments: at about 2 years, subclinical abnormalities in brain organization could become evident.

The same reason that renders the end of the second year an appropriate timepoint for identifying at-risk children also renders it the end of a period of critical importance and developmental vulnerability. Unfortunately, the period between birth and 2 years seems to be the period in which therapeutic interventions would have the greatest positive effect [25]. Data on Romanian orphanages indicate that children experiencing extreme deprivation placed in foster care before 2 years of age appear to be making far better improvements in cognitive development than those placed in foster care after age 2 [28]. The profound changes in structural brain development may delineate this critical period: paradoxically, the starting point of reliable predictive data could also be the end of the window of increased plasticity for rehabilitation. Therefore, there is a pressing clinical need to follow at-risk children very early after birth, as well as in the absence of clinical or neurological dysfunctions, and to find reliable neonatal biomarkers of risk [5, 29]. Furthermore, a greater effort should be made to implement and evaluate the effectiveness of therapeutic stimulation/support in the first two years of life of children experiencing pre-perinatal adversities.

### Supplementary Information

Below is the link to the electronic Supplementary material.

Supplementary file1 (DOCX 19 KB)

## Data Availability

Data supporting the findings of this study are available from the corresponding author on a reasonable request.

## Abbreviations

GMDS: Griffiths Mental Developmental Scales
WPPSI-III: Wechsler Preschool and Primary Scale of Intelligence III
WISC-IV: Wechsler Intelligence Scale for Children IV
