# Supplementary material for: Prospective assessment of early developmental markers and their association with neuropsychological impairment
Source: Eur J Pediatr. 2023 Sep 14;182(11):5181–9. doi: 10.1007/s00431-023-05182-y (PMC10640515; doi:10.1007/s00431-023-05182-y)
Supplement: Supplementary file 1 — Supplementary file1 (DOCX 19 KB) [file 431_2023_5182_MOESM1_ESM.docx]

**Inclusion and exclusion criteria for the study on premature infants:**

Inclusion criteria: children born < 35 gestational age.

Exclusion criteria: intrauterine growth restriction (defined as estimated fetal weight below the 10th percentile and umbilical artery pulsatility index greater than two standard deviations), craniofacial malformations, genetic syndromes, clinical evidence of neonatal encephalopathy, brain ultrasound evidence of intra-ventricular hemorrhage or periventricular cystic leukomalacia, occurrence of seizures. Furthermore, no infant being treated with drugs (e.g., sedatives) affecting the central nervous system, and no one with abnormal electroencephalogram (all electroencephalograms have been evaluated by the same person).

**Inclusion and exclusion criteria for the study on hypoxic-ischemic encephalopathy infants:**

Children suffering from hypoxic ischemic encephalopathy, and underlying therapeutic hypothermia were included following these inclusion criteria: 1) gestational age > 36 weeks, 2) any of the following: arterial umbilical cord or first blood gas analysis (within 1 postnatal hour) with pH < 7.0, and base excess <12, or 10-minute Apgar score < 5, or need for respiratory support at 10 minutes of life, 3) moderate to severe encephalopathy within 6 hours of birth. Neonatal encephalopathy was scored according to Sarnat & Sarnat criteria (Sarnat and Sarnat, 1976).

**Tables**

Table S1. Neonatal clinical characteristics of premature infants.

| **Clinical data** | **Preterm, N = 30** |
| --- | --- |
| M: F ratio | 18:12 |
| GA (days), median (IQR) | 219 (196, 234) |
| Birth Weight (g), median (IQR) | 1395 (1061, 1925) |
| Birth Length (cm), median (IQR) | 39 (35, 41) |
| Birth CC, median (IQR) | 28.1 (25.4, 30.9) |
| 1°-min Apgar score, median (IQR) | 8 (6.5, 8) |
| 5°-min Apgar score, median (IQR) | 8 (8,9) |
| pH, median (IQR) | 7.24 (7.19, 7.31) |

GA: gestational age; CC: cranial circumference; IQR: interquartile.

Table S2. Neonatal clinical characteristics of the hyoxic ischemic encephalopathy infants.

| **Clinical data** | **HIE, N = 40** |
| --- | --- |
| M: F ratio | 23:17 |
| GA (days), median (IQR) | 272 (264, 288) |
| Birth Weight (g), median (IQR) | 3280 (2979, 3695) |
| 1°-min Apgar score, median (IQR) | 4 (1, 5) |
| 5°-min Apgar score, median (IQR) | 5 (4, 7) |
| 10°-min Apgar score, median (IQR) | 6 (5, 8) |
| pH, median (IQR) | 7.4 (6.8, 7.10) |
| Base deficit [mmol/L], median (IQR) | -19 (-21, -16) |
| HCO3, median (IQR) | 17.2 (14.6, 19.9) |
| Resuscitated at birth, n (%) | 23 (57) |
| Seizures, n (%) | 4 (11) |

GA: gestational age; IQR: interquartile.

Table S3. GMDS at 12 and 24 months agreement. We fitted a linear mixed model (estimated using REML and nloptwrap optimizer) to predict the GMDS’s score with time and subtest interaction (formula: GMDS ~ time * subtest). The model included patients as random effect. The model's total explanatory power is acceptable (conditional R2 = 0.38) and the part related to the fixed effects alone (marginal R2) is of 0.11.

| **Predictors** | **Beta** | **95% CI** | **p-value** |
| --- | --- | --- | --- |
| (Intercept) | 91 | 88, 93 | <0.001 |
| Time | 0.30 | 0.21, 0.38 | <0.001 |
| *Subtest* |  |  |  |
| A | — | — |  |
| B | 6.4 | 4.0, 8.6 | <0.001 |
| C | 16 | 14, 18 | <0.001 |
| D | 18 | 16, 20 | <0.001 |
| E | 30 | 28, 32 | <0.001 |
| *time * subtest* |  |  |  |
| time * b | -0.17 | -0.29, -0.04 | 0.007 |
| time * c | -0.84 | -0.96, -0.72 | <0.001 |
| time * d | -0.84 | -0.96, -0.72 | <0.001 |
| time * e | -1.8 | -1.9, -1.6 | <0.001 |
| time * tot | -0.77 | -0.89, -0.65 | <0.001 |

**Risk factor index**

The risk factor index was calculated as follows: index was assigned: a score of 1 was allocated if at least two of the following events were reported in the in-depth interview conducted with the parents: very low socioeconomic status, mild traumatic events (such as relocation, economic problems), conflictual relationships between parents, and/or a clinically relevant score on the Parent Stress Index-Short Form. The Parent Stress Index-Short Form is a standardized tool that yields scores of parenting stress across four domains: parenting distress, difficult child, dysfunctional parent-child interaction, and total stress.

The risk factor index was otherwise scored as 0. Severe events (parental death, neglect, invalidating pathologies, or substance abuse) were considered exclusion criteria (as described in the Participant Section).
